# Photocatalytic CO_2_ Reduction Using TiO_2_-Based Photocatalysts and TiO_2_ Z-Scheme Heterojunction Composites: A Review

**DOI:** 10.3390/molecules27072069

**Published:** 2022-03-23

**Authors:** Zia Ur Rehman, Muhammad Bilal, Jianhua Hou, Faheem K. Butt, Junaid Ahmad, Saif Ali, Asif Hussain

**Affiliations:** 1 School of Physics, College of Physical Science and Technology, Yangzhou University, Yangzhou 225000, China; ziakhan4845@gmail.com (Z.U.R.); nwlbilal2@gmail.com (M.B.); asifpmesp@gmail.com (A.H.); 2 School of Environmental Science and Engineering, College of Physical Science and Technology, Yangzhou University, Yangzhou 225000, China; 3 Guangling College, Yangzhou University, Yangzhou 225009, China; 4 Jiangsu Collaborative Innovation Center for Solid Organic Waste Resource Utilization, Nanjing 210095, China; 5 Department of Physics, Division of Science and Technology, University of Education Lahore, Lahore 54000, Pakistan; junaidue5@gmail.com (J.A.); bssaifali@gmail.com (S.A.)

**Keywords:** photocatalytic activity, CO_2_ reduction, redox reactions, photocatalysts

## Abstract

Photocatalytic CO_2_ reduction is a most promising technique to capture CO_2_ and reduce it to non-fossil fuel and other valuable compounds. Today, we are facing serious environmental issues due to the usage of excessive amounts of non-renewable energy resources. In this aspect, photocatalytic CO_2_ reduction will provide us with energy-enriched compounds and help to keep our environment clean and healthy. For this purpose, various photocatalysts have been designed to obtain selective products and improve efficiency of the system. Semiconductor materials have received great attention and have showed good performances for CO_2_ reduction. Titanium dioxide has been widely explored as a photocatalyst for CO_2_ reduction among the semiconductors due to its suitable electronic/optical properties, availability at low cost, thermal stability, low toxicity, and high photoactivity. Inspired by natural photosynthesis, the artificial Z-scheme of photocatalyst is constructed to provide an easy method to enhance efficiency of CO_2_ reduction. This review covers literature in this field, particularly the studies about the photocatalytic system, TiO_2_ Z-scheme heterojunction composites, and use of transition metals for CO_2_ photoreduction. Lastly, challenges and opportunities are described to open a new era in engineering and attain good performances with semiconductor materials for photocatalytic CO_2_ reduction.

## 1. Introduction

**Figure 1:**
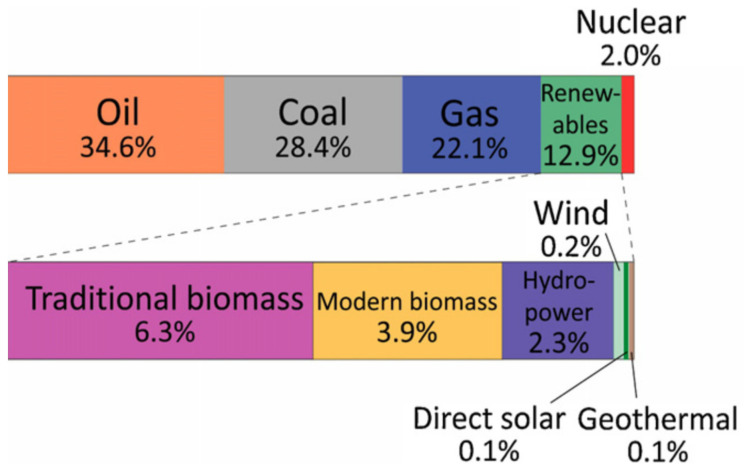
Distribution of energy consumption sources in 2008. Reprinted with permission from Elsevier, 25 December 2021 Ref. [11].

From the beginning of the 20th century, energy resources have been a major concern of human beings due to the Industrial Revolution and advancement in technology. Fossil fuels were the mainstream source of energy in that era. Consumption of fossil fuels on a large scale causes environmental pollution and increases the amount of CO_2_ in the environment. The average global temperature has increased by 1 °C in recent years due to global warming which has dangerous effects on our world [1]. CO_2_ is one of the major greenhouse gases which is produced from the burning of fossil fuel [2]. Therefore, it is necessary to reduce the emission of CO_2_ by decreasing fossil fuel usage and utilizing renewable energy (solar, water, biomass etc.) [3,4,5]. It is very important to develop systems to capture CO_2_ from the atmosphere and reduce it to useful hydrocarbon which we use as a fuel instead of fossil fuel. This technique will help to solve the global warming issue and energy shortage simultaneously [6,7,8,9,10]. The energy demand in the world is increasing day by day. A survey is done about the utilization of energy resources in 2008 [2]. The survey is described in pictorial form which is given below (Figure 1) [11]).

The sun provides us with an enormous amount of energy; the solar energy we receive in 1 h is 9200 (4.3 × 10^20^ J h^−1^) times the energy that was consumed on the earth in 1 h in 2001 (4.7 × 10^16^ J h^−1^) [12]. Researchers have conducted a lot of work to propose a mechanism to utilize solar energy. Giacomo Ciamician was an Italian photochemist; in 1912, he proposed the design of solar fuel by implementing the science of photosynthesis [13].

The reduction of CO_2_ in the presence of water by photosynthesis produces hydrocarbons and oxygen, which is solar to chemical energy conversion. The reduction of CO_2_ gives us many valuable chemicals such as methanol (CH_3_OH), methane (CH_4_), and methanol (C_2_H_5_OH) which can be used as a non-fossil fuel energy source instead of gasoline and diesel [14,15,16]. Methanol (CH_3_OH) is produced from the reduction of CO_2_ and can directly be used as fuel instead of gasoline and diesel [17]. It is not required to change the current energy bass system of the combustion engine or to make major alterations in the engine system of the motor vehicle [18]. It can be directly used as an energy source as an alternative to fossil fuel without making a change in the energy distribution system of the internal combustion engine of the motor vehicle.

In 1979, Fujishima and coworker first reported the reduction of CO_2_ by using semiconductor photocatalysts such as TiO_2_ and CdS [19]. Willner and his team purposed the photocatalyst reduction of CO_2_ to CO by using fac-Re (bpy) (CO)_3_Cl; this is selective reduction of CO_2_ to CO which has the highest quantum yield [20,21]. The researcher developed numerous semiconductor photocatalysts which have been used for this purpose such as Ti_2_O, CdS, ZnO, and Bi_2_ [22,23,24,25,26,27]. It is a difficult mechanism because it consists of multi-electron steps which lead to various useful products such as CO, CH_4_, higher hydrocarbons, alcohol, aldehydes, and carboxylic acids [28]. So, it is the main concern of researchers to study the selective photoreduction of CO_2_ to obtain the selective product.

Many efforts have been made to synthesize and modify different photocatalysts for the photocatalyst reduction of CO_2_ but their efficiency is still not enough for practical use on a commercial level. The main reasons are deficiency in the charge transfer channel and the active sites not being sufficient to fulfill the requirement for CO_2_ reduction. It is a big task to synthesize such efficient materials for CO_2_ photocatalytic reduction which have earned more light to perform better results. To resolve this problem, many techniques have been used to further activate the electronic deposition such as atomic doping and metal deposition. Some emerging concepts and platforms are facilitating the design of cocatalyst-free solar activity of TiO_2_ in a cost-effective way, with good quantum efficiency for expanding the TiO_2_’s photocatalytic applications [29].

Rutile TiO_2_ has a promising application for its effective usage of indoor illumination in photocatalytic environmental purification [30]. Single atom catalysts have gained more attention by achieving outstanding properties for CO_2_ photocatalytic reaction. These single atom catalysts can attain good utilization of prepared samples, heighten catalytic activity, and are at an economical price. Many of the single atom catalysts have been synthesized for CO_2_ reduction and display excellent results. These catalysts can also offer active sites to increase the CO_2_ photocatalytic activity. In this aspect, TiO_2_ has great potential due to its unique properties and this can play a vital role in photocatalytic CO_2_ reduction [31].

Titanium dioxide has been widely studied in material science and technology due to its functional properties and application. TiO_2_ and its composite with other materials such as graphene, g-C_3_N_4,_ and transition metal oxide have been reported for photocatalytic activity. Historically, TiO_2_ has been widely used as pigment for paints [32,33,34] and for other products such as toothpastes [35] and sunscreens [36,37,38]. A historical breakthrough took place with the discovery of photocatalytic water splitting on a TiO_2_ electrode illuminated by UV light [39], which brought about extensive interest and efforts toward the scientific and technological research on titania. In the present day, the most important applications of TiO_2_ can be roughly classified into two categories, namely environmental and energy applications. For the reasons discussed in the next sections, these applications often rely on modified forms of TiO_2_. MXene, carbon quantum dots, CdS and ZnS, and non-oxide semiconductors have exhibited excellent photocatalytic properties. The most commonly used photocatalysts have been selected from metal oxide semiconductors; some examples of them are represented by TiO_2_, ZnO, SnO_2_, CeO_2_, ZrO_2_, WO_3_, MoO_3_, Fe_2_O_3_, and Fe_2_O_3_. Among all of them, TiO_2_ has some advantages when used as a photocatalyst.

It is not expensive, while production on a large scale is cost effective.
It has excellent ability to resist corrosion as well as having good photo stability.
Many physical and chemical techniques have been well developed already to synthesize the porous film and nanoparticulate powder of TiO_2_.
It has exhibited excellent photocatalytic efficiency.
It can be activated in visible sunlight and can start a chemical reaction.

Lei Ji et al. report the direct utilization of FeP nanoarray on Ti mesh (FeP NA/TM) as an efficient 3D catalyst electrode to electrochemically convert CO_2_ to alcohols [40]. In 0.5 MKHCO_3_, the FeP NA/TM offers a high FECH_3_OH up to 80.2%, with a total FECH_3_OH+C2H_5_OH of 94.3% at −0.20 V vs. reversible hydrogen electrode (RHE). It has shown an extraordinarily high stability during 36 h of continuous electrocatalysis. Anthony Vasileff et al. report the electrocatalyst using some derived model; namely, by using iodide-derived copper (ID-Cu) and oxide-derived copper (OD-Cu) foams, they study the CO_2_ to ethane pathway [41].

After significant work has been conducted on the photoreduction of CO_2_, selectivity of the product and conversion efficiency have not improved much. To address these issues, extensive study of CO_2_ reduction processes is required, because it depends on different factors [42]. It depends on the composition of the photocatalyst and the reductant and solvent that are used in the reaction system [43].

**Figure 2:**
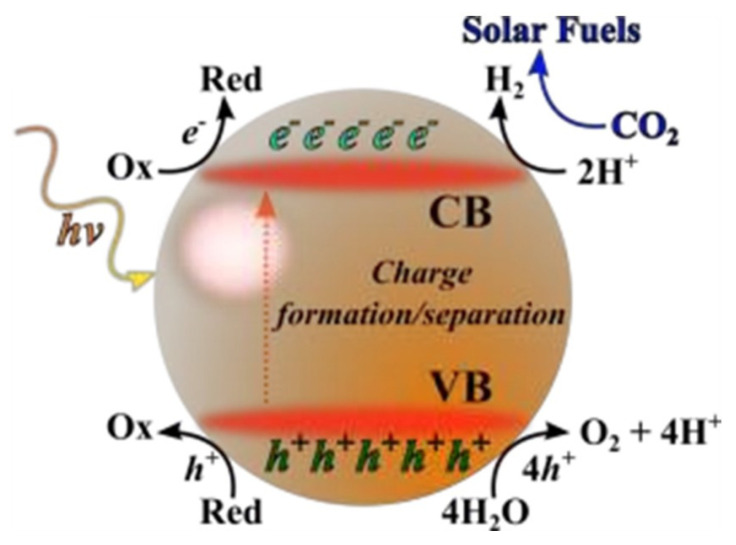
Schematic explanation of photo-induced e^−^/h^+^ pairs. Reprinted with permission from American Chemical Society, 24 December 2021. Ref. [44].

Electron (e^−^) and hole (h^+^) pairs are generated during the photocatalytic reaction in the conduction and valance band, respectively, by absorption of photon. These species then move on the surface of material to take part in the oxidation and reduction reaction. Absorption of light, generation, and separation of e^−^/h^+^ pairs and their performance in catalyzing a reaction are major factors that will define the efficiency. These three steps are explained in Figure 2.

**Figure 3:**
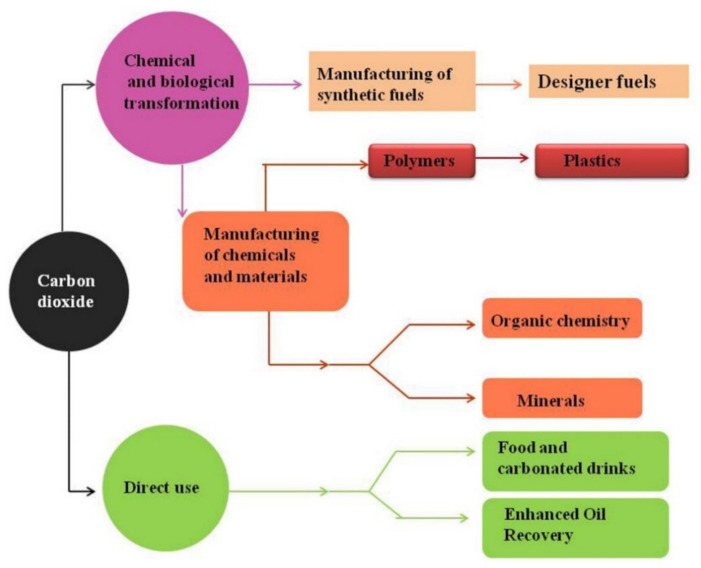
Uses of carbon dioxide on an industrial level. Reprinted with permission from Elsevier, 19 December 2021 Ref. [45].

The electronic energy band gap of the semiconductor plays the most important role in the overall process because it controls the extent of light absorption. To induce absorption of light in visible regions, different kinds of techniques have been used such as doping and usage of light sensitizers. To enhance the charge separation efficiency, the cocatalyst and Schottky junction have been used as well as doping of the correctly aligned band structure with the semiconductor employed for this purpose. There are many uses of carbon dioxide on the industrial level. We can directly use CO_2_ in food and carbonated drinks and also to increase the recovery of oil. It can also be used in the biological and chemical transformation for manufacturing synthetic fuels and also for the preparation of chemicals and materials. These materials and chemicals are helpful in the manufacturing of polymers, plastics, minerals, and in organic chemistry. Thus, we can say that CO_2_ will have more scope on an industrial level in the future. The diagram which details the uses of CO_2_ is given below (Figure 3).

## 2. The Fundamentals of Photocatalytic CO_2_ Reduction

### 2.1. Basic Principle of Photocatalysis

**Figure 4:**
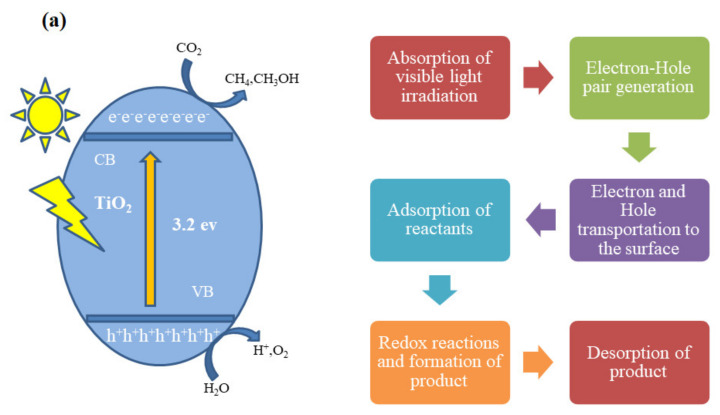

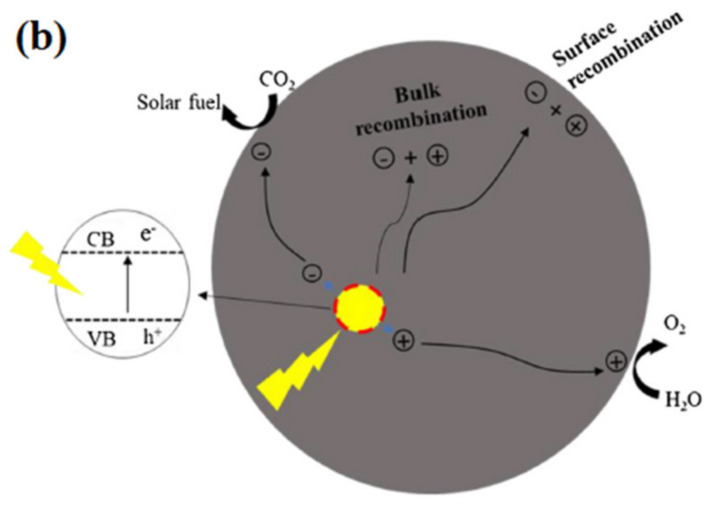
(**a**) Schematic diagram of charge recombination across photocatalyst. (**b**) Illustration of the procedure of photocatalytic CO_2_ reduction. Reprinted with permission from Elsevier, 18 December 2021 Ref. [50].

Usually, SC materials have been used as photocatalysts; when light with suitable energy falls on the catalyst, the electrons move from the valance band (VB) to the conduction band (CB). There is an energy difference between the valance band and the CB of the photocatalyst which is called the band gap [46]. The charge carriers (e^−^ and h^+^) are generated when the light strikes on the surface of photocatalysts with suitable energy greater than the band gap energy of SCs. These electrons and holes play a vital role in the oxidation and reduction reactions to obtain the product [24,47]. In this process, if photogenerated charge carriers (e^−^ and h^+^) are not rapidly exhausted, then there is the possibility to recombine and lose their energy. During this process, e^−^ and h^+^ could undergo modification on the surface of photocatalyst material and become recombined. There are two possibilities for this recombination; one is small E_BG_ of catalyst material where electrons and holes may be easily recombined and the other is where these photogenerated particles cannot find the material to react and are recombined due to a very small lifetime [48]. Some basic steps such as: (i) absorbance of light; (ii) production of e^−^ and h^+^ pairs; (iii) transportation of these photogenerated charges to the surface of the material; (iv) adsorption on the catalyst surface; (v) oxidation and reduction reactions; and (vi) formation of desired product occur during this reaction [49]. The schematic diagram of the charge recombination process across the photocatalyst material is given in Figure 4.

**Figure 5:**
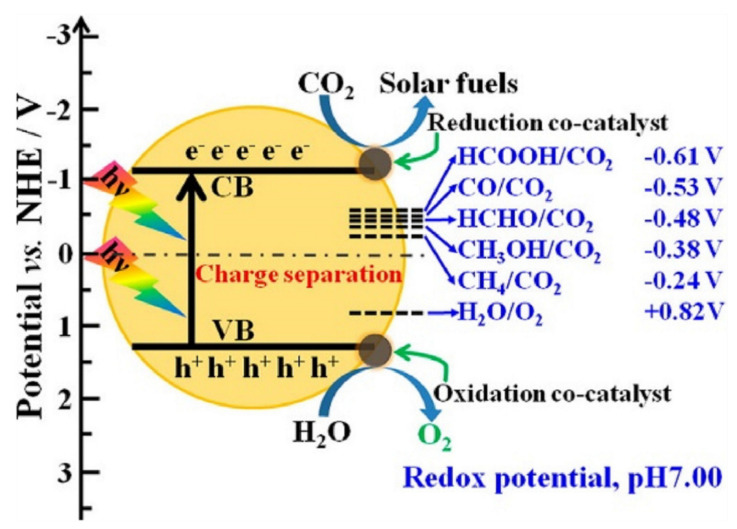
Schematic representation of a typical photoreaction process. Reprinted with permission from American Chemical Society, 20 December 2021 Ref. [52].

When light falls on material then it adsorbs according to the band gap energy of SC material and the photocatalytic activity started. This absorbed energy is used in the reductive process for the designed purpose, such as reduction of CO_2_ or removal of organic dyes from water. After the absorbance of light energy on the surface of the semiconductor, the electrons move from the valance band to the conduction and due to this, +ve charged particles (h^+^s) are produced in the valance band of the semiconductor material. This movement of charge carriers can only be possible if the band gap energy is less than the energy of the incoming light falling on the photocatalyst. The catalyst material performance and efficiency are not restricted to the band gap of material with appropriate irradiated light and inhibition (e^−^ and h^+^) recombination with different material modification methods. It also depends on many factors such as size of particle, SA, deposition process, and NHE potential of the final product. In future, to obtain better results, effectiveness of these factors and restriction of electron and hole recombination can play a significant role in photocatalytic activity [51]. The diagram of the typical photoreaction process for the production of different renewable fuels is given below in Figure 5.

**Figure 6:**
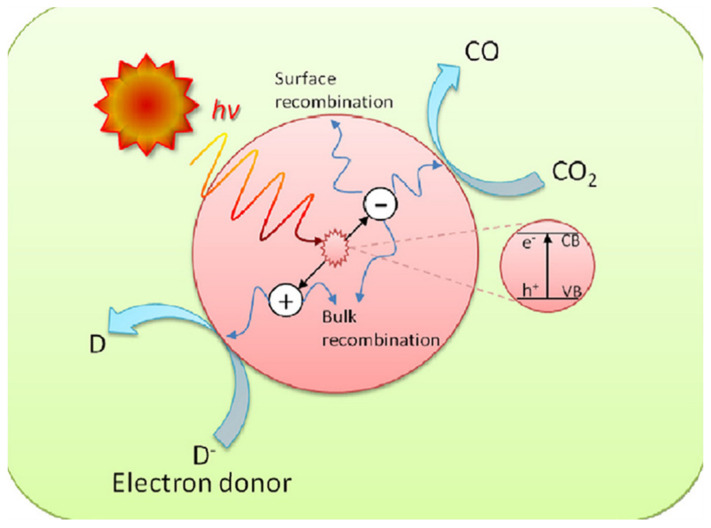
Schematic representation of photoexcitation and electron transfer process. Reprinted with permission from Elsevier, 18 December 2021 Ref. [49].

In the photocatalytic process, when light strikes with the material highly active, charge carriers are produced for reaction. In the valance band, the uppermost energy band is filled with electrons, whereas in the conduction band there are no or very few electrons at its ground state. For the reduction reaction it is needed for CB to have more negative than the required potential [53]. In the opposite site the oxidation process occurs due to the conduction band electrons reducing the absorbed species; however, valence band electrons oxidize these species through surface-bound hydroxyl radicals [54]. Normally, these electrons move on the surface of material and combine at the trapping areas then react with species such as CO_2_ [55]. If the CR is slower than the transition reaction, then this process can only occur. When the electrons flop to search the trapped places or use those materials which have small band gap energy, then the recombination process happens in the photocatalytic reaction. If recombination occurs, then small amounts of energy are released at the surface of material [49]. A schematic diagram of photo-excitation and electron transformation is given below in Figure 6.

**Figure 7:**
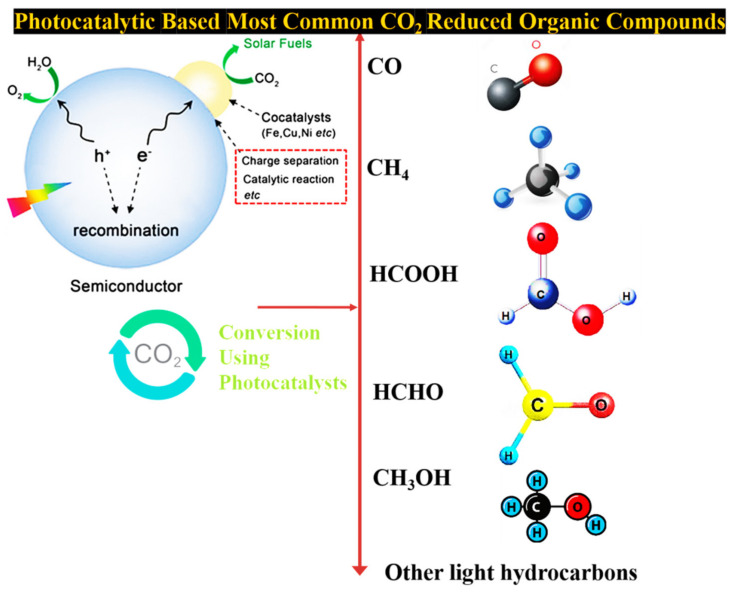
Mechanism of photocatalytic reduction of CO_2_ and the most common reduced organic compounds produced in this reaction.

The possible photocatalytic CO_2_ conversion mechanism commonly includes three main phases: (I) photogenerated electron–hole pairs; (II) separation and transformation on the surface of catalysts; and (III) redox reaction among photogenerated charge carriers and surface adsorbed species. The CO_2_ reduction was achieved in both solution and gas phase. The most common reduced organic compounds are shown in Figure 7.

**Figure 8:**
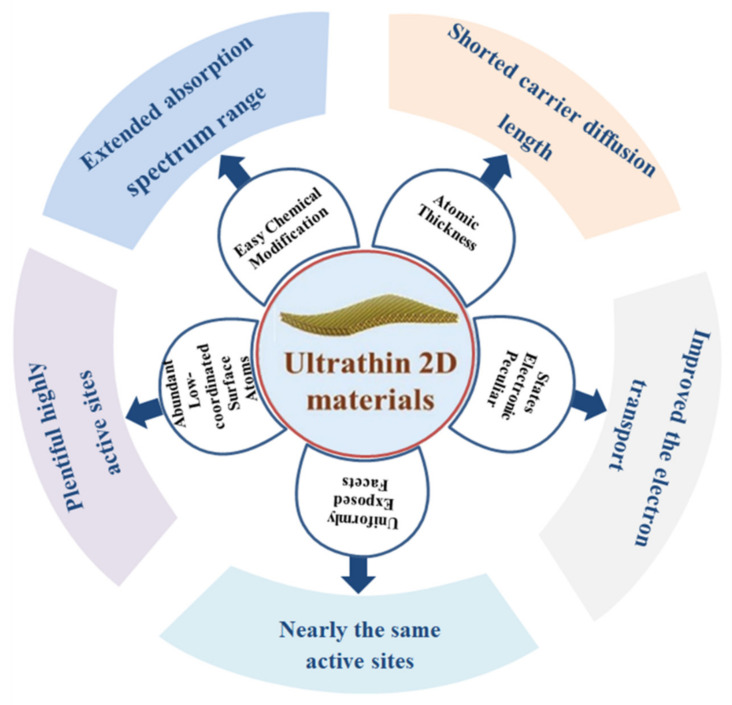
Diagram showing the benefits of ultrathin 2D materials. Reprinted with permission from Royal Society of Chemistry, 19 December 2021 Ref. [57].

The photocatalytic CO_2_ reduction has to face two challenges: one is thermodynamics and the other is kinetics. The CO_2_ molecule has large bonding energy which is 750 kJ mol^−1^. The CO_2_ reduction is required to absorb an appropriate amount of light which is necessary to break the double carbon oxygen bond (C=O). The breaking of the C=O bond is essential to form the C-H bond and this bond is the reason for developing the hydrocarbon chemical. The oxidation and reduction reactions with expectations of CO_2_ and water are difficult reactions which require more environmental energy compared to the water splitting reaction which is sometimes the reason for low photocatalytic activity. When we use semiconductor photocatalysts, some problems are faced in the energy band structure which affects the absorption of photons or photocatalytic activity. So there is a need to improve these factors and obtain better efficiency for photocatalytic CO_2_ reduction [56]. To reduce these problems, we have synthesized many 2D materials which may be useful for CO_2_ reduction. In this aspect, ultrathin 2D materials have become an attractive candidate due to their exclusive properties for photocatalytic CO_2_ reduction. Figure 8 reveals the advantages of ultrathin 2D nanomaterials.

### 2.2. Thermodynamics of Photocatalytic Reduction of CO_2_

The thermodynamics in photocatalytic activity for CO_2_ reduction can be described with some factors such as temperature, light which falls on the photocatalyst, and CB and VB of semiconductor. Commonly, the SCs we use in the photocatalytic reaction have fully filled VB and incompletely filled or empty CB. The energy levels present in the atom have different quantities of photogenerated charge carriers [50]. Some factors which are mostly effective on CO_2_ photoreduction are given below.

#### 2.2.1. Effect of Temperature

When light falls on the SC material with energy >E_g_, then it varies the quantity of electrons and holes in the CB and valance band, respectively. Electrons attain equilibrium within a few seconds in the energy level of CB as compared to band gap energy because relaxation time is small in CB [58,59]. The thermodynamic force is used to start the photocatalytic activity and is in direct relation to the difference of the e^−^ and h^+^ quantity in the CB and VB, respectively [60]. Through experimental work, it is proven that heat cannot induce the photocatalytic phenomenon but the output of CO_2_ reduction via the photocatalytic reaction rises with temperature [61]. In this process, temperature helps in the desorption of product from the photocatalyst surface that increases the carbon dioxide reduction yield [62]. Thus, the escalation in the temperature is helpful in the photocatalytic process to increase the rate of reaction.

#### 2.2.2. Effect of Light

In the photocatalytic process, light radiation plays an important role in starting the process because when light falls on the SC material, excitation occurs and electron hole pairs are generated. The chemical potential also helps to elucidate the process because chemical potential of e^−^ is greater in quasi Fermi level than h^+^ potential and is consequently ΔG positive. So, the light radiation gives necessary ΔG and starts the photocatalytic reduction of CO_2_ at normal temperature. Thus, it is proven that thermodynamics of this photocatalytic reaction may be explained by the free energy system with the help of light radiation.

**Figure 9:**
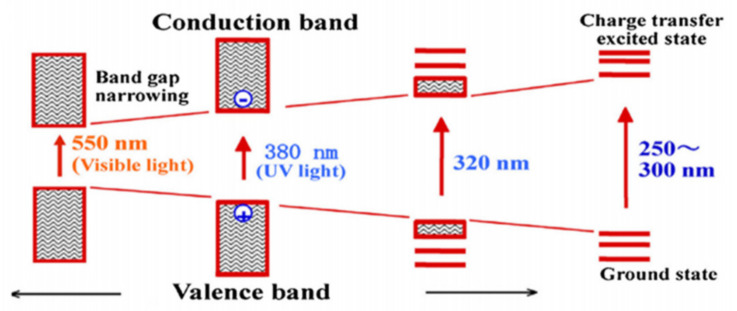
Narrowing and expansion of band gap of semiconductor and wavelength of incoming light. Reprinted with permission from Elsevier, 19 December 2021 Ref. [66].

When we discuss the energy aspect, then photocatalytic reduction of CO_2_ primarily depends on the intensity and ʎ of light. The wavelength of light is important for the excited e^−^ and h^+^ energy because it depends on ʎ of light. The quantity of e^−^ and h^+^ in the conduction band is due to the intensity of falling light [63]. If we consider UV light with high-energy photons, then it can generate e^−^ and h^+^ pairs in the large energy band gap of SC [64]. The band gap and ʎ of light should correspond with one another. The high-energy photon such as for UV light is necessary to initiate the stimulation of large band gap SCs. As compared to large band gap materials, the smaller band gap photocatalysts are activated with the help of visible light (>380 nm) [65]. Similarly, the light with high power intensity can produce a large number of e^−^ and h^+^ pairs when it falls on the surface of the photocatalyst. Thus, it is proven that the efficiency of CO_2_ reduction via photocatalytic activity rises with intensity of light and decreases with escalation in the ʎ of light [66]. However, sometimes in the presence of high power intensity light, results are not so good due to rapid recombination of e^−^ and h^+^ in photocatalytic reduction of CO_2_. The band gap of SC materials and the incoming light wavelength are important to each other to obtain better performance [65]. The diagram which details the relation between ʎ of light and the band gap of semiconductor materials is given below in Figure 9.

#### 2.2.3. Effect of CB and VB Potential

**Figure 10:**
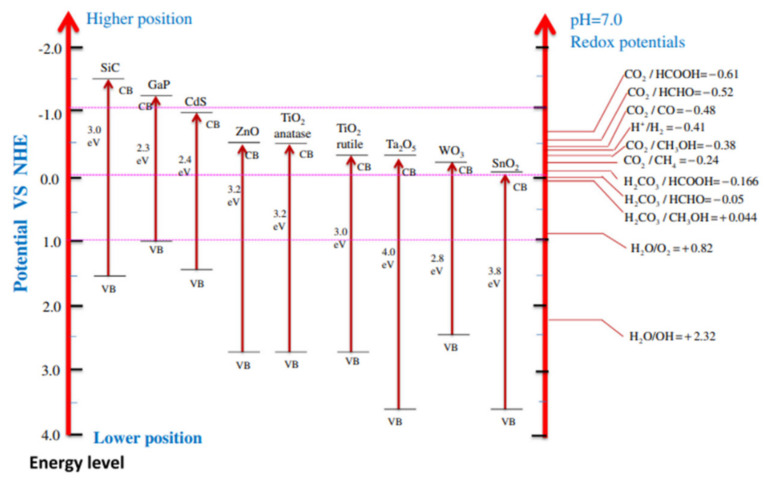
Schematic diagram of CB potentials of semiconductors and thermodynamic reduction potentials of various photocatalyst measured at pH 7. Reprinted with permission from Elsevier, 19 December 2021 Ref. [67].

In the photoreduction of CO_2_, two types of reactions are involved: one is CO_2_ reduction and the other is oxidation of H_2_O. Usually, we consider the thermodynamics of CO_2_ reduction; then we also explain it in respect to CB and VB of the photocatalyst. The possibility of thermodynamics for the production of renewable and green fuels can be determined with the help of the position of CB in the photocatalyst material. The position of CB, the VB of various photocatalysts, their band gap energy and reduction potential related with promising green and renewable fuels are described in Figure 10. We take an example of TiO_2_ with a valence band and conduction band potential of 2.7 eV and −0.50 eV, respectively, as the photocatalyst for CO_2_ reduction. If we discuss this thermodynamically, then some environmentally friendly fuel may be formed which has less (-ve) reduction potential than the conduction band of TiO_2_ from carbon dioxide photoreduction such as CH_4_ (−0.24 eV) and CH_3_OH (−0.38 eV). Similarly, TiO_2_ photocatalyst can oxidize those materials which have less redox potential than its valence band potential [67]. Through results, it is proven that CB, VB, and energy obtained from the light play a significant role in thermodynamics of photocatalytic CO_2_ reduction.

### 2.3. Recombination of Charges and Effect of Metal-Modified Surface

**Figure 11:**
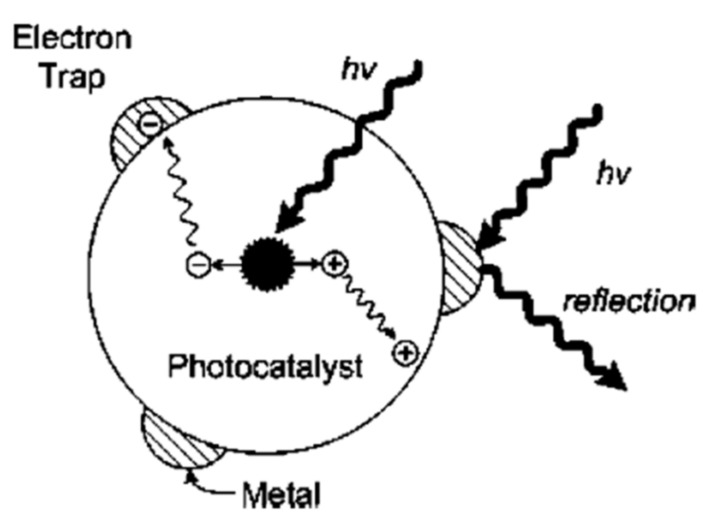
Metal-modified surface photocatalyst. Reprinted with permission from American Chemical Society, 16 December 2021 Ref. [73].

The recombination of e^−^ and h^+^ is a big problem in the efficiency of photocatalytic activity of CO_2_ reduction. Many research articles have been written on the photocatalysts with metal loading in the photocatalytic CO_2_ reduction process. These photocatalysts have been used as charge carrier traps to increase the efficiency of the photocatalytic reaction. Those materials which have this property have the ability to overcome the recombination of e^−^ and h^+^ pairs and can raise the lifetime of these photogenerated charge carriers. This phenomenon is described in Figure 11 which is given below. Many scientists have published papers on the performance of metal-modifying photocatalysts. When Cu/TiO_2_ is used as a photocatalyst in the CO_2_ reduction process for CH_4_, then it is observed that the layer of copper (Cu) on the surface of the photocatalyst helps in the higher efficiency of photocatalytic activity [68]. Similarly, the composites of Hg and Pt with TiO_2_ also showed good performance for formaldehyde [69]. Some other composites of metals such as Pd, Rh, Au, and Ru with TiO_2_ have given better performance for the production of methane and acidic acid [70]. In the above given metals, the combination of Pd/TiO_2_ was the promising material, and researchers have usually selected this for the production of methane through the CO_2_ reduction process [71]. In 1987, Thampi et al. described how rhodium/TiO_2_ showed a good performance for photocatalytic CO_2_ reduction in the existence of hydrogen [72].

From the above given metal composites, it is observed that when more electrons are available, the efficiency of CO_2_ photoreduction for the production of CH_4_, methanol, and formaldehyde remarkably increased. The reason is that when metal atoms make contact with the SC surface, the e^−^ easily comes from the semiconductor (SC) and smoothly spreads on the surface of the material. Moreover, the oxidation process can happen because in metal/TiO_2_ composites, holes are easily diffused to the SC surface. In these composites, two things are important for the photocatalytic CO_2_ reduction process: one is that metals are uniform and equally distributed over the surface of the photocatalyst. The other is that large amounts of metal loading create problems in the illumination of the catalyst surface due to the reflection and large amounts of photons cannot be absorbed [73].

## 3. Synthesis Methods of TiO_2_-Based Photocatalyst

**Figure 12:**
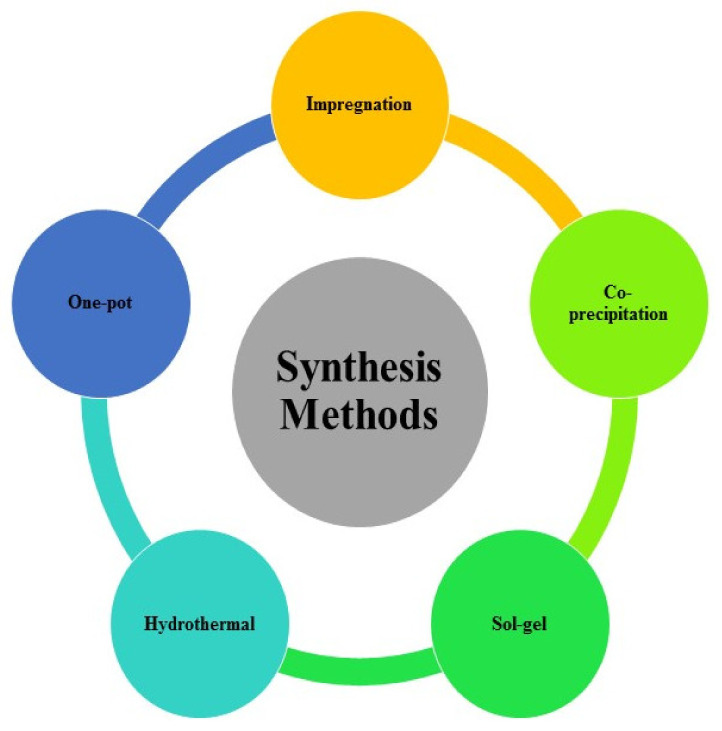
Different synthesis methods for the preparation of materials for CO_2_ reduction.

In this section, we discuss various synthesis methods such as hydrothermal, sol-gel, impregnation, one-pot, co-precipitation, and other methods. Figure 12 illustrates some possible synthesis techniques.

### 3.1. Sol-Gel Synthesis

Xia et al. [74] developed the MWCNT and TiO_2_ composites by utilizing the sol-gel technique for the reduction of CO_2_ with H_2_O. They synthesized the nanocomposites by coating the anatase TiO_2_ on the MWCNT. The existence of MWCNT in the synthesized composite can reduce the accumulation of the particles of TiO_2_ and move the electron hole pairs developed due to the irradiation of UV light and can mitigate the electron hole pair recombination and consequently enhance the photocatalytic performance of TiO_2_. The synthesized nanocomposite materials lead to the major development of C_2_H_5_OH. It was observed that the MWCNT shows excellent performance for TiO_2_ as compared to the activated carbon. The synthesized nanocomposite showed higher performance as compared to the pure TiO_2_ in the reusing cycles.

**Figure 13:**
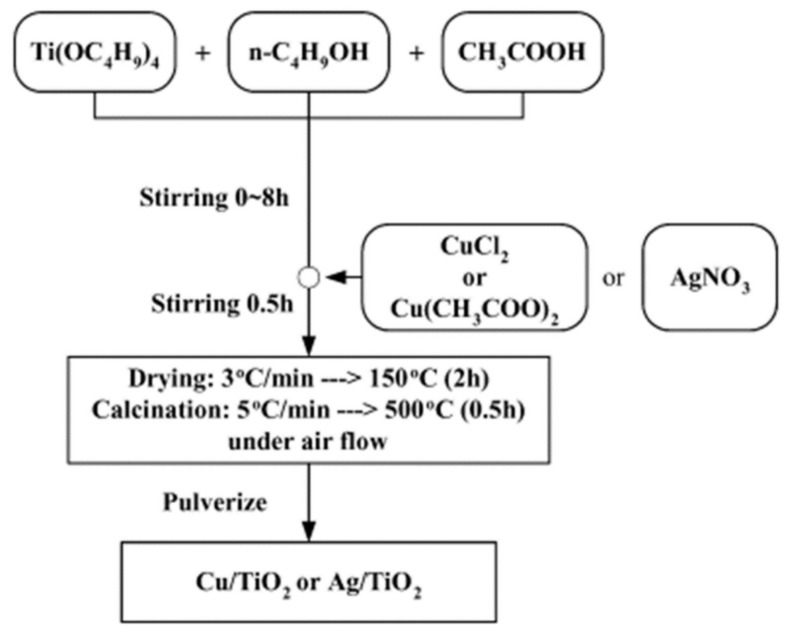
Sol-gel process for the development of the catalysts. Reprinted with permission from Elsevier, 19 December 2021 Ref. [75].

IH Tseng and JCS Wu [75] fabricated the Cu/TiO_2_ and Ag/TiO_2_ catalysts by using the modified sol-gel approach as shown in Figure 13. They evaluated the reduction of CO_2_ and observed that the division of Cu in the particles of TiO_2_ is crucial to enhance the production of methanol. A maximum value of methanol production was ~1000 μmol g_catalyst_^−1^ with the 25 molar% of complete loading of Cu on the layer of the particles of TiO_2_. The photocatalytic performance of the Ag/TiO_2_ was lower as compared to the Cu/TiO_2_ owing to the vigorous affinity among the clusters of Ag and photoelectrons [75]. Nishimura et al. [76] also fabricated the films of Cr-doped TiO_2_ by using the sol-gel approach to improve the CO_2_ reduction.

### 3.2. Co-Precipitation Method

**Figure 14:**
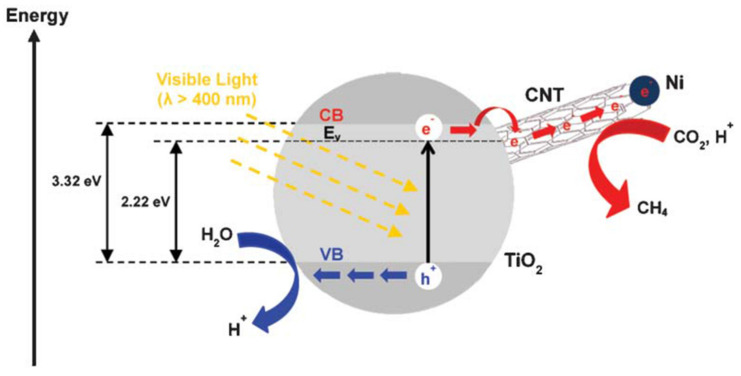
Charge transfer mechanism for the reduction of CO_2_ with H_2_O. Reprinted with permission from Royal Society of Chemistry, 20 December 2021 Ref. [77].

Ong et al., [77] successfully fabricated the CNT@Ni/TiO_2_ photocatalyst for the reduction of CO_2_ into CH_4_ under the irradiation of visible light. They synthesized the nanocomposites by utilizing the co-precipitation technique. The results showed that the synthesized nanocomposites have band gap of 2.22 eV, due to which they can absorb a large amount of energy from visible light. The photocatalytic performance revealed that the fabricated composite showed maximum CH_4_ production (~0.145 μmol g_catalyst_^−1^ h^−1^) as compared with pristine TiO_2_ and Ni/TiO_2_. This improvement was due to the collaborative interaction among the CNTs and TiO_2_. Figure 14 illustrates the charge transfer procedure for the reduction of CO_2_.

### 3.3. Impregnation Method

Gui et al. [78] successfully developed the MWCNT@TiO_2_ by doping them with metal oxides by utilizing the early wetness impregnation approach. The nanocomposites doped with CuO, CoO, and Fe_2_O_3_ played a pivotal role in the expansion of the absorption band into the visible light portion and also facilitated the charge separation. Among the developed nanocomposites, CuO-MWCNT@TiO_2_ showed an excellent photocatalytic performance and production of CH_4_ was recorded as 0.93 μmol g_catalyst_^−1^ at the eighth hour of irradiation time. They also proposed the mechanism of charge transfer for these synthesized metal-oxide-doped MWCNT@TiO_2_ nanocomposites for the reduction of CO_2_ [78].

Kohno et al. [79] fabricated the nanocomposites of Rh and TiO_2_ by using the facile impregnation technique for the reduction of CO_2_ with hydrogen. The reaction started in the absence of light but it was improved under irradiation, and was considered as a “photo-enhanced reaction”. When Rh in the nanocomposites of Rh/TiO_2_ was turned down into a complete metallic condition, the performance was reduced and the major product was converted from CO to CH_4_. The same behavior was also observed by raising the loading amount of Rh.

### 3.4. Hydrothermal Synthesis

Xia et al. [74] fabricated the MWCNT and TiO_2_ composites by employing the hydrothermal technique for the reduction of CO_2_ with H_2_O. They developed the nanocomposites by the uniform deposition of rutile TiO_2_ nanorods on the MWCNT. The existence of MWCNT in the synthesized composite can reduce the accumulation of the particles of TiO_2_ and move the electron hole pairs developed due to the irradiation of UV light and can mitigate the electron hole pair recombination and consequently enhance the photocatalytic performance of TiO_2_. The synthesized nanocomposite materials lead to the major development of HCOOH. It was observed that the MWCNT shows excellent performance for TiO_2_ as compared to the activated carbon.

### 3.5. One-Pot Synthesis

**Figure 15:**
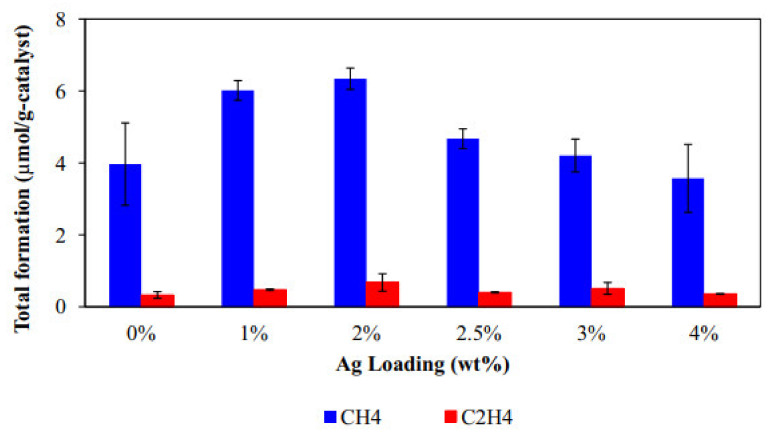
Production of CH_4_ and C_2_H_4_ over Ag-MWCNT@TiO_2_ for various concentrations of Ag. Reprinted with permission from Elsevier, 19 December 2021 Ref. [80].

Gui et al. [80] successfully fabricated the Ag-MWCNT@TiO_2_ nanocomposite by using the simple one-pot synthesis approach for the reduction of CO_2_. Different concentrations of Ag were used to observe the effect on the reduction of CO_2_. Among all the samples, the sample having 2% concentration of Ag was considered the ideal sample that showed the maximum reduction of CO_2_ into C_2_H_4_ and CH_4_ with the values of ca. 0.68 μmol g_catalyst_^−1^ and 6.34 μmol g_catalyst_^−1^, respectively, as shown in Figure 15. The maximum value of CH_4_ production was recorded as ca. 0.91 μmol g_catalyst_^−1^ h^−1^ with the 2% concentration of Ag. The observed values were 2.06- and 1.06-fold higher as compared to the undoped MWCNT@TiO_2_. The improved results were attributed to the loading of Ag that enhances the electron-hole pair recombination rate that is the potential factor for the photocatalytic reactions.

### 3.6. Other Methods

Gui et al. [81] fabricated the core-shell nanocomposites of MWCNT and TiO_2_ for the reduction of CO_2_ under the irradiation of visible light. The nanocomposites were synthesized by utilizing the new coating technique. UV-Vis evaluation showed that the synthesized nanocomposites revealed improved photocatalytic performance under visible light. The synthesized nanocomposites showed continuous conversion of CO_2_ into CH_4_ under the irradiation of low visible light at atmospheric pressure. The maximum production of CH_4_ was recorded as ca. 0.17 μmol g_catalyst_^−1^ h^−1^ at the sixth hour of irradiation time [81].

## 4. TiO_2_-Based Photocatalysts for CO_2_ Reduction

Semiconductors are useful for CO_2_ reduction. Titanium dioxide is widely explored for photocatalysts for CO_2_ reduction among the semiconductors due to its suitable electronic/optical qualities, availability at low cost, thermal stability, low toxicity, and high photoactivity. Anatase, rutile, and brookite are three different polymorph structures of TiO_2_. Anatase phase has the highest photoconductivity and has a band gap of 3.2 eV [82,83]. The photoreactivity of the rutile phase is lower than the other two phases due to its greater proficiency in recombination of the electron-hole pairs and smaller surface area. However, it has limited efficiency due to its wider band gap of 3.2 eV; after absorption of UV photons, it produces low oxygen, and the charge transport property is relatively poor. As it is active in the UV region, it is thus limited due to UV photosensitivity because 3 to 5 percent of UV light reaches the earth’s surface. So, that is the reason that it should be photosensitive under visible light. For TiO_2_, high energy is required for activation but in UV light only 4% of photons have high energy. This is the main reason TiO_2_ has limitations for photocatalytic activity. To eliminate these limitations, researchers are working on various modification techniques for the TiO_2_ surface, such as dye sensitization, doping, synthesis of composite semiconductors, quantum dots, modification using metal oxides, the formation of nanomaterials, modification by surface photosensitization, and formation of heterostructures [84]. A detailed survey of different TiO_2_-based photocatalysts for CO_2_ reduction is summarized in Table 1, where major products produced, reactants, temperature, and pressure for TiO_2_ photocatalysts are mentioned.

### 4.1. Doping

The most common method to modify the catalytic properties to tune the band gap of TiO_2_ is doping. As metals are electron-rich, they can behave as electron-trapping agents. Efficiency of TiO_2_ and charge separation properties can be enhanced and improved by metal doping. At the interface of the metal-semiconductor, a strong electric field is created when TiO_2_ is doped. Numbers of charge carriers are increased at the interface by the produced electric field. So, these charge carriers move toward the surface where they can be used in a reduction reaction. Therefore, doping for the reduction of CO_2_ is a successful and easy method and improvement of the photocatalytic efficiency of TiO_2_ can be achieved. So, we can dope TiO_2_ with metals and non-metals. The process of doping can be carried out by using various methods such as the sol-gel, metal-ion implantation [85], co-precipitation method [86], impregnation, and microwave-assisted method [84,87].

#### 4.1.1. Metal-Doped TiO_2_ Photocatalysts

When a TiO_2_ is doped with metals, then electrical properties can be adjusted or modified by the impurities of metal in the pure structure of TiO_2_. The band gap of pure TiO_2_ is 3.2 eV, which is large enough that it is incapable of absorbing high-energy photons. So, the band gap is decreased with the doping of metals. Doping of TiO_2_ has been conducted by different researchers with metals, noble metals, rare-earth metals, and transition metals.

**Figure 16:**
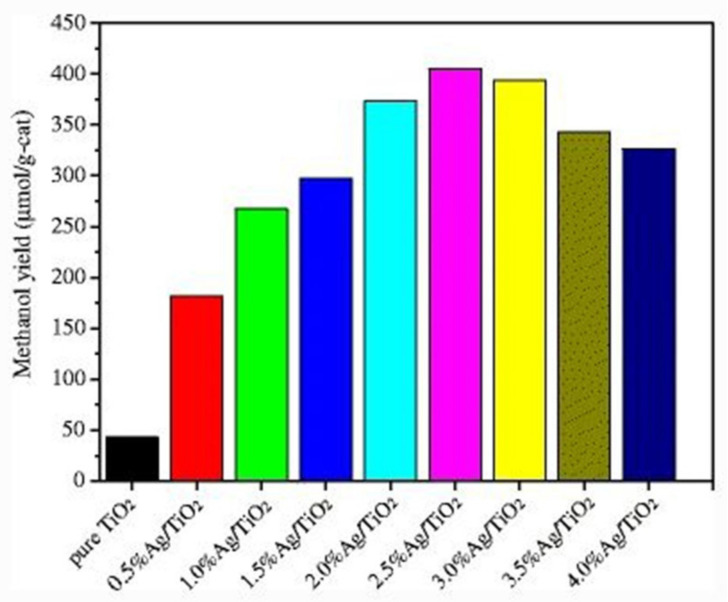
Ag/TiO_2_-based photocatalysts for reduction of CO_2_, with different samples showing higher methanol yield than pure TiO_2_. Reprinted with permission from Springer Nature, 21 December 2021 Ref. [87].

Metals such as Ag, Cr, Cu, Rh, Fe, and Pt are commonly used to make metal-doped TiO_2_-based photocatalysts for CO_2_ reduction. Enzhou et al. successfully prepared Ag/TiO_2_ nanocomposites for reduction of CO_2_ via the microwave-assisted method by doping different percentages of Ag. The composite with 2.5% Ag/TiO_2_ showed the best performance as a photocatalyst under visible light and UV that was 9.4 times higher and better than pure TiO_2_. It produced methanol that yielded 405.2 μmol/g-cat (Figure 16) [87].

The pure TiO_2_ structure is doped with transition metals to modify its band gap due to which the heteroatom-doped photocatalyst is formed and on the doped metal atom, the photoconversion of CO_2_ takes place. This photoconversion occurs when electrons are transferred from the valance band (VB) to the conduction band (CB) and then to the heteroatom of the metal.

At the metal-semiconductor interface, the Schottky barrier is formed, when the n-type semiconductor (TiO_2_) is doped with the metal, due to which holes cannot migrate from semiconductor to metal. This is because of work function; the work function of metal W_m_ is greater than the work function of the semiconductor (W_s_). To overcome this problem, the work function of the semiconductor (W_s_) should be greater than the work function of metal [43]. Charge carriers can easily migrate in the case of p-type semiconductors (e.g., Cu_2_O). The choice of dopant metal is crucial to know because it determines the pathway of the photocatalyst reaction and type of the product. When TiO_2_ is doped with Pt, it produces methane from CO_2_ which has higher yield than methanol. The reason for this is that the work function of Pt is greater than the work function of TiO_2_, due to which photogenerated electrons can easily migrate or be transferred. Among all the metals that are used for CO_2_ reduction, copper Cu and silver Ag are the most frequently used metals that showed higher TiO_2_ photocatalytic activity and higher selectivity of methanol production in CO_2_ reduction.

#### 4.1.2. Non-Metal-Doped TiO_2_ Photocatalysts

**Figure 17:**
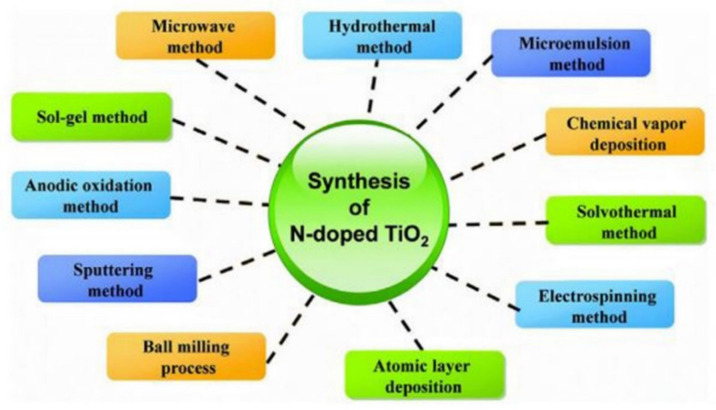
Different methods that are suitable to prepare nitrogen-doped TiO_2_. Reprinted with permission from Elsevier, 19 December 2021 Ref. [84].

Doping with non-metals like C, B, S, N, and P, etc. is a good method to modify the TiO_2_ photocatalytic system. Nitrogen is the most famous and frequently used candidate by the researchers for doping with TiO_2_. Because of its atomic size that is similar to oxygen, low ionization energy, and high stability, it is used to narrow the band gap. Under visible light, Kumar et al. used the nitrogen doping for CO_2_ reduction into methanol. They used nitrogen-doped graphene supported on the copper complex. They reported that during the photocatalytic process, nitrogen helped to reduce recombination. Different methods have been reported by the researchers to synthesis the nitrogen-doped TiO_2_. Figure 17 shows the different methods for the TiO_2_ doped with nitrogen.

On the other hand, phosphorus is the least used non-metal for the photoreduction of CO_2_ with TiO_2_. Wang et al. described the P-doped TiO_2_ nanotubes for photoreduction of CO_2_. They reported that high methanol yield was achieved that was up to 860.4 μmolg/cat.

#### 4.1.3. Defect Chemistry of Doped TiO_2_

When defects occur in semiconductor-based photocatalysts, their performance can be greatly impacted. There are always defects in semiconductors, which significantly affect the charge separation and transport processes. In semiconductors, localized states that are due to defects can selectively capture approaching charge carriers, resulting in improved photocatalytic activity. Alternatively, these defect states can work as centers of recombination for charge carriers. In addition, the defect states also influence charge transport, altering free carrier acceleration vectors and generating potential barriers. To improve the efficiency and effectiveness of charge separation and photocatalysis, a clear understanding of the effects of defects is required [88]. The photoactivity of the composite catalyst is determined by the crystallinity of the composite catalyst, the crystal phase, and defects. There have been several methods for tuning TiO_2_ surfaces by introducing defects into the forbidden gap. Adding cations to either the interstitial or substitutional sites of TiO_2_ causes a change in density of states near Fermi levels [89].

**Figure 18:**
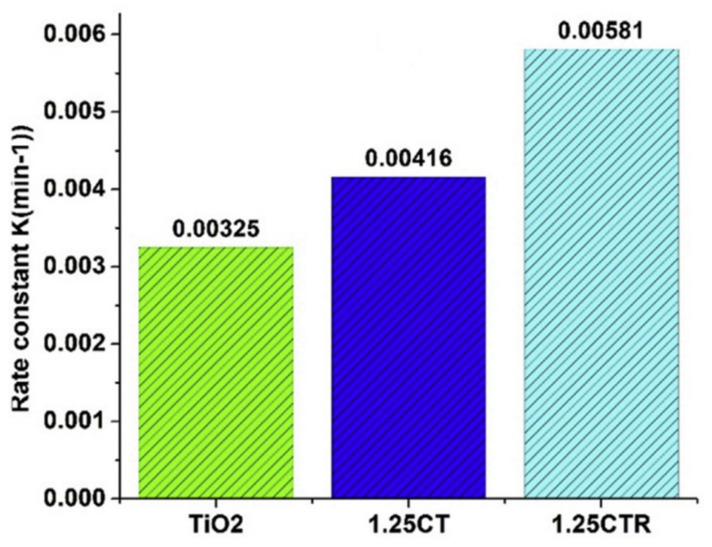
Effect of defects on rate constant with different ratios of Ce and Ti. Reprinted with permission from Elsevier, 19 December 2021 Ref. [89].

Dileep Maarisetty et al. studied the defect chemistry of Ce-doped TiO_2_. In order to enhance the interfacial contact between the oxides of Ce and Ti, they introduced defects on the surface of the composite catalyst by thermal treatment. The rate constant of Ce-doped TiO_2_ increased by 40% under solar light by thermal treatment in an inert atmosphere at 900 °C compared to catalysts of the same composition annealed at an alternative heating condition (Figure 18) [89].

### 4.2. Modification Using Metal Oxides (MO)

Pure TiO_2_ is also doped with metal oxides because metal oxides improve the light absorption, surface chemistry, structural properties, and charge separation of the photocatalysts that give the higher photocatalytic activity. Charge separation is achieved at the heterojunction of MO and TiO_2_, due to which the redox reaction is separated which is helpful to obtain more product yield from the CO_2_ reduction. Due to the smaller band gap of MO, the light absorption capacity of TiO_2_ is improved. Slamet et al. reported methanol product in the photoreduction process of CuO that gave the highest yield. Cu_2_O is very promising and is the most frequently used dopant candidate for the photoreduction of CO_2_. It has a band gap ranging from 2 to 2.2 eV that absorbs visible light effectively.

**Figure 19:**
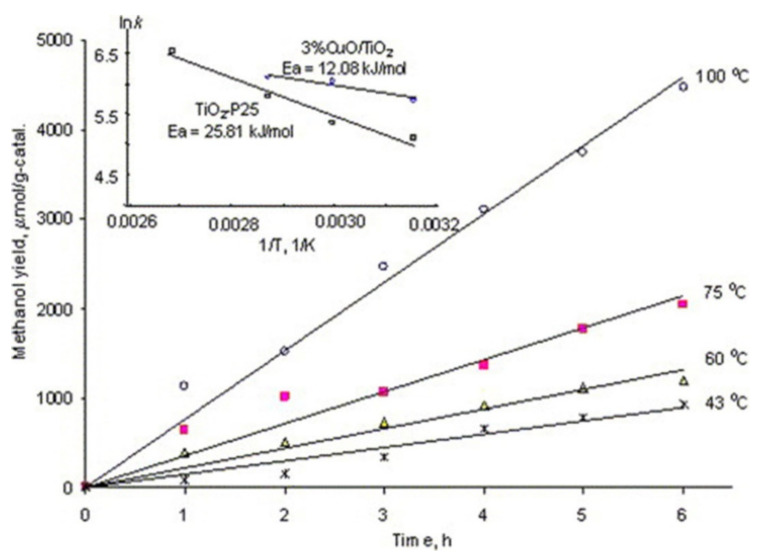
Temperature dependence of methanol production on TiO_2_, plot of TiO_2_, and 3% CuO/TiO_2_ photocatalysts. Reprinted with permission from Elsevier, 19 December 2021 Ref. [90].

Nasution et al. also reported the methanol production when TiO_2_ is doped with CuO. The synthesis method they used was the improved impregnation method. The light source they used was a black light lamp with UV of 10 W with 415 nm to 700 nm wavelength and 2.45 mW cm^2^. The production of methanol was 442.5 and 19.23 3 μmolh^−1^ per g-cat. In Figure 19, a typical Arrhenius plot for 3% CuO/TiO_2_ catalysts and Degussa P25 is shown [90].

### 4.3. Formation of Nanomaterials

Nanostructured nanomaterials have various advantages in the photocatalytic process due to their faster diffusion rate and high surface area. The recombination between electrons and holes is inhibited. The surface to volume ratios is high for nanomaterials, so they perform good catalytic activity. Nanostructured nanomaterials have been widely researched due to their advantages. Scientists successfully synthesized the materials that have nanostructure such as nanofibers, nanorods, nanostrings, core-shell, nanowires, and ribbons. Yolk-shell, multi-shell, and hollow shells are also nanostructured nanomaterials that are known as heterostructured nanomaterials. These nanostructures improve the photocatalytic response of the photocatalyst. The abovementioned properties and morphologies of nanomaterials and nanocomposites result in well-defined pathways for electrons and these electrons are interconnected so that their transport rate becomes rapid and wider. These nanostructures help to inhibit recombination and allow separation of electron-hole pairs effectively [84,91].

**Figure 20:**
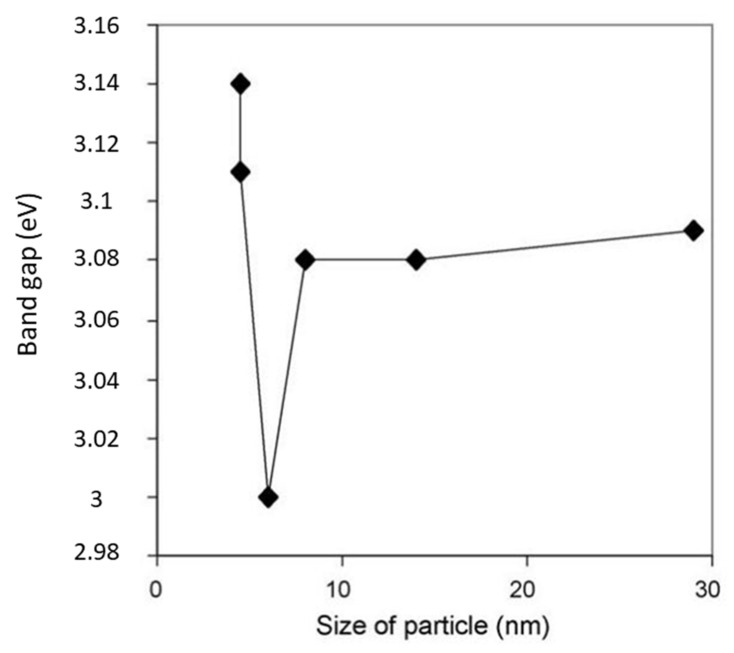
Effect of particle size on band gap. Reprinted with permission from Elsevier, 21 December 2021 Ref. [84].

The spatial electronic configuration of carbon nanotubes and 1D nanowires shows brilliant results. Wang et al. investigated the nanowires of Pt-TiO_2_ and stated that enhanced photocatalytic activity and these nanowires have properties like graphene that were more effective for separation of electron hole pairs [92]. Limited works exist that describe the importance of heterostructure nanomaterials such as core-shell structure as reported by Tsai et al.; Dai et al. reported flower-like structure; Junior et al. and Ijaz et al. reported rod-like structure; and Hefeng et al. reported hollow structures that have enhanced photocatalytic response [93,94,95,96,97].The particle size has effect on band gap which is given below in pictorial form (Figure 20).

### 4.4. Crystal Phases

**Figure 21:**
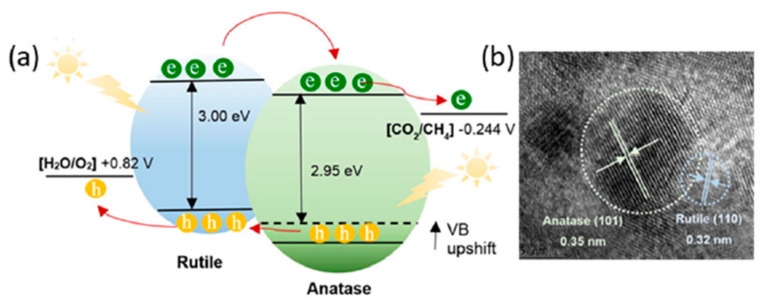
(**a**) Heterojunctions induced from rutile and anatase crystal phases of TiO_2_. (**b**) TEM images of anatase and rutile phases. Reprinted with permission from American Chemical Society, 22 December 2021 Ref. [101].

TiO_2_ has three kinds of crystal phases: anatase, rutile, and brookite. For photocatalytic activity, the first two phases are usually reported. By creating the mixture of its different phases, its photocatalytic performance can be improved [98,99,100]. For this purpose, Chai et al. reported the controlled composition of the anatase and rutile phase in TiO_2_ to improve its photoactivity by simply varying the annealing temperature. They found that TiO_2_ nanoparticles were composed of both anatase and rutile crystal phase, the heterojunction of which leads to an increase in electron-hole separation. Morphology is another factor that can affect the photoactive ability of TiO_2_. To make sure these hypotheses are clear, 1D hierarchical mesoporous TiO_2_ nanofibers were synthesized using a combination of electrospinning and sol-gel methods (Figure 21) [101].

### 4.5. Modification by Surface Photosensitization

**Figure 22:**
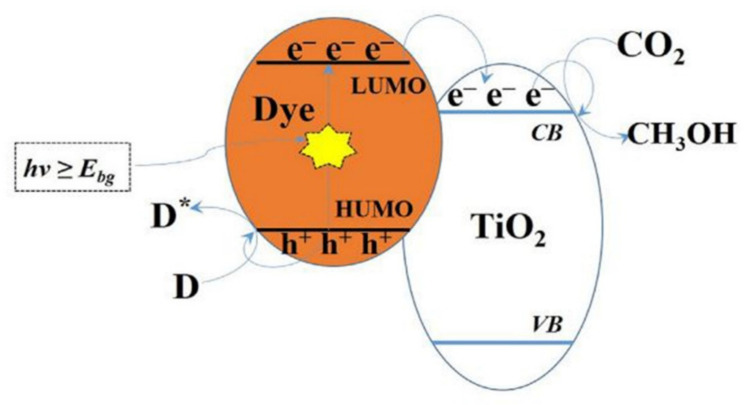
Photoreduction scheme of CO_2_ to methanol using dye-sensitized TiO_2_ photocatalyst. Reprinted with permission from Elsevier, 19 December 2021 Ref. [84].

Surface photosensitization expands the useable light wavelength and the efficiency of the excitation process is increased. The absorption rate of visible light can be enhanced by using sensitizers. Sensitizers such as dye sensitizers are ones that are used widely. Figure 22 illustrates the photoreduction scheme of CO_2_ by using dye-sensitized TiO_2_ photocatalyst into methanol. In this system, dye served as the light harvester and the TiO_2_ photocatalysts as electron accepter. The process begins like other photocatalytic systems and photoexcitation occurs. The photoexcitation of electrons occurs from the highest occupied molecular orbital (HOMO) of the dye to its lowest unoccupied molecular orbital (LUMO), which is followed by the photogenerated electron transference from the LUMO of the dye to CB of TiO_2_ and then it is used up by CO_2_ reactant. Then it is converted into hydrocarbons. The main advantage of this method is the possibility of recycling the oxidized dye through oxidation of H_2_O. The photosensitive dye provides photoelectrons that are in contact with the semiconductor material. At the surface between an electrolyte, semiconductor and dye charge separation phenomenon occurs. The excitation process is increased by the surface photosensitization through chemisorbed or physiosorbed dye that expands the wavelength of useable light through excitation. Numerous research works have used this technique for reduction of CO_2_. However, the efficiency of CO_2_ conversion into other hydrocarbons and methane is minimal [102,103,104].

A comparison of the different TiO_2_-based photocatalysts for CO_2_ reduction is given in Table 1, where reactants and temperature and pressure, major products produced, and reactor type are presented for TiO_2_ photocatalysts.

**Table 1:** Summary of photocatalytic CO_2_ reduction literature over various types of TiO_2_-based photocatalysts.

Photocatalyst Name	Reactants		T, P	Light Source	Major Product: (Formation Rate µmol h^−1^ g_car_^−1^)	Reactor Type	Ref.
TiO_2_ Powder	CO_2_	H_2_O	P = 6.5 MPa	990 W, Xe Lamp	HCOOH	High-pressure Stainless-steel Vessel, Pyrex cell H-shaped	[105]
Cu/TiO_2_	CO_2_	H_2_O	T = 273–323 K, P = 1 atm	75 W, High-Pressure Mercury Lamp, λ > 280 nm	CH_4_, CH_3_OH, CO	Quartz cell	[106]
Cu/TiO_2_	CO_2_	H_2_O	25 °C, 1 atm	450 W, Xe-lamp	Ethylene, Methane	Stainless steel with quartz window	[107]
TiO_2_	CO_2_	H_2_O	NA	Hg Lamp, UV light, Ultrahigh pressure, 500 W, λ = 350 nm	CH_4_	Slurry Reactor	[108]
TiO_2_	CO_2_	H_2_O	273 K	8 W, λ = 254 nm, Hg lamp	CH_4_	Quartz tube Reactor	[109]
Thin film, Pt (1 nm)-TiO_2_	Mixture CO_2_	H_2_O vapor	NA	400 W, Xe lamp, (250–388 nm)	CH_4_: 1361	Flow Reactor System	[110]
CuO-TiO_2_ (hollow microspheres)	CO_2_	H_2_O	P = 3.45 bar	Hg Lamp, 40 W, 254 nm	CO: 14.54, CH_4_: 2.07	Closed System	[111]
TiO_2_ (P25)	Saturated CO_2_	H_2_O	T = 278 K	HP Hg arc, 500 W	CO: 0.35	Photo-Kolbe reaction of acetic acid	[112]
Au/TiO_2_	CO_2_	H_2_O	P = 2 bar	6 W lamp	CH_4_: 8.0	Steel Reactor	[113]
Fe/TiO_2_	CO_2_	H_2_ + H_2_O	343 K	Xe Lamp	CO: 8.2	Cylindrical Vessel Reactor	[114]
Pd/TiO_2_	CO_2_	H_2_	NA	150 W, Hg lamp	CH_4_: 355.6, CO: 46.3, C_2_H_6_: 39.6	Miniature Visual Autoclave	[115]
Pd/TiO_2_	CO_2_	H_2_O	NA	500 W Hg lamp	CH_4_:1.415, CO: 0.722	Stainless steel chamber	[116]
Ag/TiO_2_	CO_2_	H_2_O	25 °C, 1 atm	8 W Hg Lamp	CH_3_OH: 9.0, CH_4_: 8.5	Stirred batch annular slurry reactor	[117]
TiO_2_/zeolite	CO_2_	H_2_O	P = 10^−6^ torr	75 W high-pressure lamp	CH_4_	Quartz cell with a flat bottom	[106]
MoS_2_/TiO_2_ nanosheets	--	H_2_O	P = Atmosphere	300 W Xe lamp	CH_3_OH: 10.6	Airtight quartz glass reactor	[118]
MoS_2_/TiO_2_ fibers	--	H_2_O	NA	350 W Xe lamp	CH_4_: 2.86	Homemade apparatus	[97]
2D/2D SnS_2_/TiO_2_	--	H_2_O	P = 1 MPa	300 W Xe lamp	CH_4_:23	50 mL stainless steel reactor with quartz flakes	[119]
CdS/TiO_2_ film	---	H_2_O	NA	300 W Xe lamp	CH_4_: 11.9	Under simulated sunlight irradiation (from a 300 W Xenon arc lamp)	[120]
CdS(Bi_2_S_3_)/TiO_2_	--	H_2_O	NA	500 W Xe lamp with cutoff Filter	CH_3_OH	Continuous-flow reactor	[121]
ZnIn_2_S_4_/TiO_2_ nanobelts	--	H_2_O	NA	300 W Xe lamp	CH_4_: 1.135	Gas tight system	[122]
CuGaS_2_/RGO/TiO_2_	--	H_2_O	P = 1 atm	300 W Xe lamp	CO: 0.15	Batch-type top irradiation cell with a Pyrex window	[123]

## 5. TiO_2_ Z-Scheme Heterojunction Composites for CO_2_ Photoreduction

Heterojunction engineering is observed to be a very encouraging technique for suppression of charge recombination as well as for obtaining applicable conduction and valance band edge positions for enhanced photocatalytic activity [124,125]. Particularly, the Z-scheme approach, encouraged with the natural photosynthesis process, promotes excellent redox facilities that would be advantageous for holes and radicals generation and propagation [124,126,127]. Fabricating Z-scheme between TiO_2_ and other semiconductors having higher potential of the conduction band is considered to be a challenging task for CO_2_. Such that, g-C3N4 CB edge is about −1.23 eV greater than TiO_2_ (−0.50 eV) potential energy; however, g-C3N4/TiO_2_ Z-scheme composite is synthesized under UV-visible to facilitate charge carrier separation [128,129,130]. For visible light irradiation, WO_3_/TiO_2_ Z-scheme heterojunction composites with suppressed charge recombination are considered to be an encouraging combination [131,132]. Raza. A et al. successfully synthesized WO_3_-TiO_2_/Cu_2_ZnSnS_4_ Z-scheme heterojunction with CO/CH_4_ yield rates of 15.37/1.69 μmol h^−1^ g^−1^ for CO_2_ production [133].

**Figure 23:**
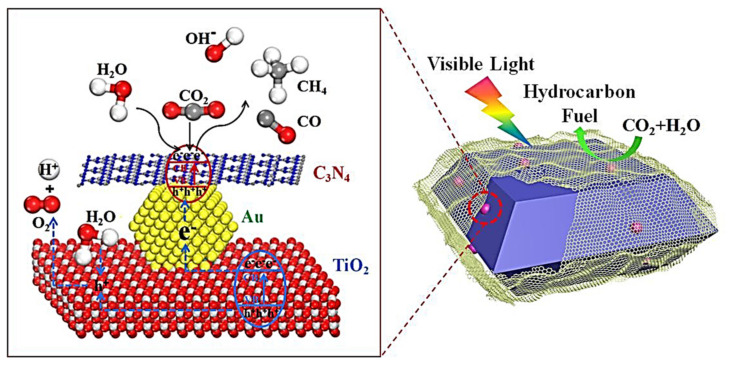
Efficient Z-scheme photocatalysts of ultrathin g-C_3_N_4_-wrapped Au/TiO_2_ nanocrystals for enhanced visible-light-driven conversion of CO_2_ with H_2_O. Reprinted with permission from Elsevier, 19 December 2021 Ref. [137].

Moreover, surface engineering of photogenerated electrons and absorbing reactants is very important to enhance the yield rate of products; the fact of the matter is that approximately eight electrons are needed for CO_2_ photoreduction to CH_4_ [134]. It is well understood that the noble metal Ag supports the surface plasmonic effects, which could be triggered by nanoparticles, and is effective in increasing the catalytic activity [135,136]. In the same way, CO_2_ photoreduction conversion into CH_4_ and CO, with the yield rate 37.4 and 21.7 μmol g^−1^ h^−1^, respectively, was achieved using (Au/A-TiO_2_)@g-C3N4 Z-scheme heterojunction [137]. The proposed mechanism is shown in Figure 23. The CO_2_ photoreduction signifies a promising route for transformation into hydrocarbon fuels using light irradiation. Currently, the productivity of CO_2_ photoreduction is significantly poor when it comes to fulfilling practical demands. Furthermore, the production of particular products of CO_2_ photoreduction is still a challenge, which will require advanced investigation in upcoming studies.

## 6. Challenges and Recommendations

Photocatalytic CO_2_ reduction has become an attractive area of research because it provides a green and environmentally friendly process to produce renewable fuels. By using these renewable fuels, we can reduce the use of fossil fuels and increase the potential to utilize low-carbon fuels for sustainable development in the future. The factors which can affect the performance of CO_2_ photoreduction are surface affinity, separation of charge carriers, and the ability of the materials to absorb light. Surface affinity suppresses the side reaction, which is important for selectivity and output of the process. If we raise the basic sites on the surface, then we can improve the surface affinity. The capacity of materials for the absorption of light and charge separation capability are related to the quantum yields and product yields [138].

The main challenges in the photocatalytic CO_2_ reduction are improvement in e^−^ and h^+^ pair separation, light absorbance efficiency, and increment in the productive yields. So, in the future perspective, many effective and supportive methods are projected to remarkably increase in the photocatalytic activity of CO_2_ reduction [139].

The greenhouse gases and the increasing requirement of energy are also large challenges from the photochemical point of view. In this aspect, many appropriate SCs with narrow band gap have been investigated to reduce these problems. The process of photocatalytic CO_2_ reduction is not an easy and simple method. There are some complications in the kinetic and thermodynamic process; due to this, CO_2_ photoreduction is not yet suitable on an industrial level. During the past few years, many research articles have been written and useful results have been obtained with different suitable photocatalyst materials. There is another problem while using present materials because they have some limitations in photocatalytic CO_2_ reduction.

In this aspect, TiO_2_ has been researched many times, but its large band gap and its low light absorption capability are hurdles in good performance. To reduce these problems, many methods have been used but have not yet obtained desirable results. Some techniques such as doping and making of heterostuctures have given better results. Many researchers have also investigated and used different methods to increase the efficiency of TiO_2_ catalyst. TiO_2_ has many properties such as the following: it has good stability, is environmentally friendly, has easy availability, and its reuse ability is better as compared to the other SC materials, but it is less photoactive. So there is a need to find those materials which are more active for light, stable, and suitable for the photocatalytic CO_2_ reduction. These materials should be active in the visible light region, and should decrease the recombination rate, improve the charge separation ability, and be suitable for CO_2_ photoreduction. In this aspect, some materials such as g-C_3_N_4_, graphene, and GO, etc. have been used for CO_2_ reduction but there is need to improve and some modifications are needed for making suitable candidates for photocatalytic CO_2_ reduction. Now, many researchers are working on the heterostuctures and these structures may play a significant role in increasing the productivity of CO_2_ reduction. Thus, there is a need to find some novel materials and progressive methods to increase the photocatalytic activity of CO_2_ reduction [84].

Those materials which are used in CO_2_ reduction define rate of reactant (CO_2_) adsorption, recombination rate, flow of e^−^ and h^+^, rate of desorption, and in reality the product of this process. Therefore, it is required to improve the present catalyst materials or find some new materials to obtain a better performance. Moreover, the information about reaction mechanism, reaction paths, and kinetic and thermodynamic studies for CO_2_ reduction is still not sufficient. The intensity of falling light and absorbance rate are important for the photocatalytic activity of CO_2_ reduction. The only way to increase the efficiency of photocatalytic reduction of CO_2_ is to understand the mechanism of CO_2_ reduction, how recombination occurs, and how to adjust these materials for a better product. Furthermore, more investigations in the field of photochemistry and surface interaction will give a better understanding about actions on the surface of materials and also give better guidelines about the selection of materials. For this purpose, simulations and innovative characterization methods can extend our knowledge to obtain better results.

Many research articles have been written on CO_2_ photoreduction and semiconductors such as TiO_2_ have become an attractive candidate to obtain creditable results. In spite of recorded attainments many SCs for photoreduction still face some problems such as low thermal and chemical stability, small absorbance of light, and low efficiency under specific circumstances. On the other hand, polymeric materials show attractive properties such as the following: being economical in price, low toxicity, easy availability, light in weight, easy to use and synthesize, and having good flexibility [140]. g-C_3_N_4_ is one of the attractive polymeric photocatalysts which showed better results even without composite of noble metals. The polymer materials for photoreduction have many properties such as good absorbance of light in VLR, porosity in structure, and appropriate band gap materials. Many of them have good mobility of charge carriers and long lifetime of excited charges [141]. In polymer materials, the generation of e^−^ and h^+^ pairs is faster than the SC photocatalysts such as in g-C_3_N_4_ due to stacked π bonds [142]. These collected π bonds in the polymeric materials are helpful in the fast charge transformation. Thus, due to the unique and distinct properties of polymeric materials, they are suitable candidates for the photocatalytic activity of CO_2_ reduction [142,143]. These stacked π bonds can play a vital role in the photoreduction of CO_2_ but the study is in the beginning stage for π-conjugated materials [140].

Increasing research into the C-based polymeric materials may be more helpful for the photocatalytic CO_2_ reduction as compared to the present SC photocatalysts. Although these promising materials have revealed good results, there is need for some modifications and suitable band gaps to obtain the best results. The design of materials and reaction methods is important but the performance and sustainability of the process for energy production and conversion also have great importance. When we discuss energy production and conversion, the life cycle of the device will also have more importance in future perspectives. So, there is need for more detailed and extensive study about photocatalytic CO_2_ reduction reactions using different and advanced tools. These strategies will give a complete and sustainable assessment about photocatalytic activity of CO_2_ reduction for the fabrication of renewable fuels and will solve the greenhouse gas effect in future perspectives [144].

## 7. Conclusions

The basic cause of global warming is the emission of CO_2_ which is escalating day by day due to the burning of fossil fuels in excessive amounts to fulfill the energy demands. The most environmentally friendly and economical technology for sustainable development is photocatalysis. So, the reduction of CO_2_ into various environmentally friendly products is a gratifying solution to reduce and utilize the emitted CO_2_. It is a good approach to sustain societies through generation of renewable energy. Titanium dioxide (TiO_2_) is widely explored for photocatalysts for CO_2_ reduction among the semiconductors due to its suitable electronic/optical qualities, availability at low cost, thermal stability, low toxicity, and high photoactivity. In this review article, photoreduction of CO_2_ has been discussed by using the TiO_2_-based photocatalysts. A detailed survey of TiO_2_-based photocatalysts, composites with TiO_2_, and metal- and non-metal-loaded TiO_2_-based photocatalysts has been presented. Moreover, the Z-scheme heterojunction composites for CO_2_ photoreduction have been studied and it was concluded that they provide an easy way to enhance efficiency of CO_2_ reduction. Various suitable and environmentally friendly synthesis methods to prepare photocatalysts for CO_2_ reduction into green products have also been studied and discussed in detail. In future, several effective and helpful methodologies are expected to be developed which will increase the efficiency of CO_2_ photoreduction and production rate.
